# Cranial Endocast of the Lambeosaurine Hadrosaurid *Amurosaurus riabinini* from the Amur Region, Russia

**DOI:** 10.1371/journal.pone.0078899

**Published:** 2013-11-13

**Authors:** Pascaline Lauters, Martine Vercauteren, Yuri L. Bolotsky, Pascal Godefroit

**Affiliations:** 1 Department of Palaeontology, Royal Belgian Institute of Natural Sciences, Bruxelles, Belgium; 2 Service d’Anthropologie et Génétique Humaine, Université Libre de Bruxelles, Bruxelles, Belgium; 3 Geological and Nature Exploration Institute, Far Eastern Branch of the Russian Academy of Sciences, Blagoveschensk, Russia; University of Birmingham, United Kingdom

## Abstract

Information on the structure of the brain of the lambeosaurine hadrosaurid dinosaur *Amurosaurus riabinini*, from the Late Maastrichtian of Blagoveschensk, Far Eastern Russia, is presented based on endocranial casts. The endocasts are compared with physical and digital endocasts of other dinosaurs. The olfactory tract was large. The cerebral hemispheres are enlarged and round, illustrating the important development of this part of the brain in hadrosaurids. The pituitary body is enlarged as well, perhaps prefiguring the large size attained by hadrosaurids. The EQ of *Amurosaurus* was similar to that of the lambeosaurine dinosaur *Hypacrosaurus altispinus* and was relatively larger than in most extant non-avian reptiles, including sauropod and ceratopsian dinosaurs. However, it was apparently relatively smaller than those of most theropod dinosaurs. The relatively large size of the cerebrum is consistent with the range and complexity of social behaviors inferred for lambeosaurine dinosaurs.

## Introduction

Since 1902, and the first discovery of dinosaur fossils in the Amur region (Far Eastern Russia), thousands of bones were collected from this area. Most of them belong to hadrosaurid taxa. *Amurosaurus riabinini* Bolotsky and Kurzanov, 1991 [1], [2] and *Kerberosaurus manakini* Bolotsky and Godefroit, 2004 [3] were found in Blagoveschensk, whereas *Olorotitan arharensis* Godefroit, Bolotsky, and Alifanov, 2003 [4] was found in Kundur, both localities being in the Amur Region of Far Eastern Russia. *Charonosaurus jiayinensis* Godefroit, Zan, and Jin, 2000 [5], [6], *Sahaliyania elunchunorum* Godefroit, Shulin, Tinghai, and Lauters, 2008 [7] and *Wulagasaurus dongi* Godefroit, Shulin, Tinghai, and Lauters, 2008 [7] were found in the adjacent region of China (Heilongjiang Province).

Among this material, some braincases of *Amurosaurus* were found. The brain is a structure very sensitive to rapid decay after the death of the animal and is thus generally lost before any fossilization. Natural endocasts are rare [8], [9], and until recently the endocranial cavity of fossil taxa was generally inaccessible for study without destructive preparation ([10]: p. 38). Recently, high-resolution x-ray CT scan was often used to access the endocranial cavity of fossil specimens. However, in some cases this method is impractical; the specimen may be too small or too large to be CT scanned, or researchers cannot access easily the machine or the cost to use it is too high. It is also possible in some cases to make an endocast without damaging the specimen, using materials such as silicone or latex rubber [11], [12].

The purpose of this paper is to describe the endocast and the cranial nerves of *Amurosaurus riabinini*, and is based on the assumption that the casts provide a good insight into the general morphology of the brain [13], [14], [15], [16], [17]. Subsequently, we compare the encephalization quotient and the cerebral volume of *Amurosaurus* to those of other dinosaurs. Independently published data about the endocranial anatomy of this species based upon the same material [18] is also discussed, and interpretations contrasted with those presented here. Finally, hypotheses about the behavior of lambeosaurine hadrosaurid dinosaurs are proposed.

## Materials and Methods

### Institutional Abbreviations

AEHM, Amur Natural History Museum, of the Amur Complex Integrated Research Institute of the Far Eastern Branch of the Russian Academy of Sciences, Blagoveschensk, Russia (Amur KNII FEB RAS); IRSNB, Institut Royal des Sciences Naturelles de Belgique, Brussels, Belgium.

### Specimens

IRSNB R 279 (endocast of AEHM 1/232), IRSNB R 280 (endocast of AEHM 1/233), and IRSNB R 281 (endocast of AEHM 1/90), and AEHM 1/240.

### Ethics Statement

This study is based on study of material held in the collections of the Amur Natural History Museum. No permits were required for the described study, which complied with all relevant regulations.

### Description

The casts were obtained on the basis of a complete and undistorted braincase (AEHM 1/232) and partial braincases (AEHM 1/90 and AEHM 1/233) of *Amurosaurus riabinini*. AEHM 1/232 and AEHM 1/90 were described in the revision of the genus by Godefroit et al. [2]. The specimen AEHM 1/233 shows the prominent median process between the basipterygoid processes typical of *Amurosaurus riabinini* and is referred to this taxon. The presence of valleculae was observed on an additional specimen (AEHM 1/240). The complete braincase (AEHM 1/232) was used to make the cast IRSNB R 279 and the description of the global features of the endocranial cavity. More details about the cranial nerves could be observed on the casts of two partial braincases: IRSNB R 280 and IRSNB R 281. These specimens were collected during the 1980s field campaign from the Upper Cretaceous Udurchukan Formation (Maastrichtian, Late Cretaceous) by Yuri L. Bolotsky. The braincases are fused and from large, presumably adult, individuals. For a complete description of the braincase of this species, see Godefroit et al. [2]. For general measurements of the braincase AEHM 1/232, see Table 1. The specimens used here were also among those used by Saveliev et al. [18] in their independent study of the endocranial anatomy of this species. However, this study is based on a different set of casts and new interpretations about the endocranial anatomy and possible behavior of *Amurosaurus* are presented.

**Table 1:** General measurements of the braincase AEHM 1/232.

Frontal length	93
Frontal width	63 (left)
Parietal length	99
Occipital condyle width	59

Measurements given in mm.

The specimens were molded using the following technique. The complete braincase was prepared by covering the smallest foramina and fractures with modeling clay, the foramen magnum was left free. A thin layer of Vaseline was sprayed into the endocranial cavity to prevent excessive adherence of the silicone. Silicone was mixed with a catalyst and poured into the endocranial cavity to create the first endocranial silicone layer. This first layer was allowed to dry for at least 24 hours. Once dry, additional silicone was poured at intervals to create a multi-layered cast. This technique allows the strengthening of the endocranial cast and prevents its tearing. When the last layer of silicone was completely dry, the endocranial cast was pulled out and the braincase was subsequently cleaned.

The volume of the endocast was measured by placing it, beforehand filled with tiny glass marbles, in water and measuring water displacement.

### Encephalization Quotient

The encephalization quotient (EQ) is an estimation of the relative size of the brain and represents the actual brain size of an individual divided by the expected brain size for its particular body size calculated using an allometric relationship derived from a large extant sample [19], [20]. According to Jerison [19] and Hopson [13], there is a negative allometry in vertebrates between brain size and body size. Based upon EQ, Jerison [19] noted that living vertebrates cluster into two groups: endotherms and ectotherms. Hopson [13] concluded that the EQs of dinosaurs are usually placed between those of modern ectotherms and endotherms. Hurlburt [14] adapted Jerison’s “lower” vertebrate equation for non-avian-reptiles and defined a Reptile Encephalization Quotient (REQ). REQ = M_Br_/(0.0155*M_Bd_^0.553^), where M_Br_ is the mass of the brain (in grams), and M_Bd_ is the mass of the body (in grams). The mass of the brain is obtained by multiplying the volume of the brain by 1.036 g/ml [21].

In extinct taxa, both the brain and body masses must be estimated, leading to many uncertainties in the calculation of the REQ. Given that no complete skeleton of *Amurosaurus* has been discovered, it is difficult to estimate the mass of an adult individual, because no braincase is directly associated to appendicular bones. Because AEHM 1/232 clearly belongs to a large adult specimen, we selected the longest femur and humerus and measured their circumference in order to estimate the mass of a large adult *Amurosaurus*. The specimens were selected according to their lambeosaurine characteristics [2] and for their size indicating that they belonged to an adult individual [22]. In addition, the ratio of humerus/femur circumferences of the selected bones matches the ratio obtained for articulated adult hadrosaurid skeletons [23], [24]. REQ calculations for dinosaurs usually estimated the volume of the brain under the assumption that the brain occupied 50% of the endocranial volume [14], [20], [25]. According to Evans [17] and Evans et al. [26], the extensive valleculae in hadrosaurids imply that the brain occupied a larger portion of the endocranial cavity than in other ornithischians and they calculated the REQ based on a brain size estimate of 60% endocast volume. Because valleculae can also be observed in *Amurosaurus* specimens, we here follow the assumption of Evans et al. [26].

## Results

### Systematic Paleontology

Dinosauria Owen, 1842 [27]

Ornithischia Seeley, 1887 [28]

Ornithopoda Marsh, 1881 [29]

Ankylopollexia Sereno, 1986 [30]

Hadrosauriformes Sereno, 1986 [30]

Hadrosauroidea Cope, 1869 [31]

Hadrosauridae Cope, 1869 [31]

Lambeosaurinae Parks, 1923 [32]

*Amurosaurus* Bolotsky and Kurzanov, 1991 [1]

*Amurosaurus riabinini* Bolotsky and Kurzanov, 1991 [1]

### Description

**Figure 1:**
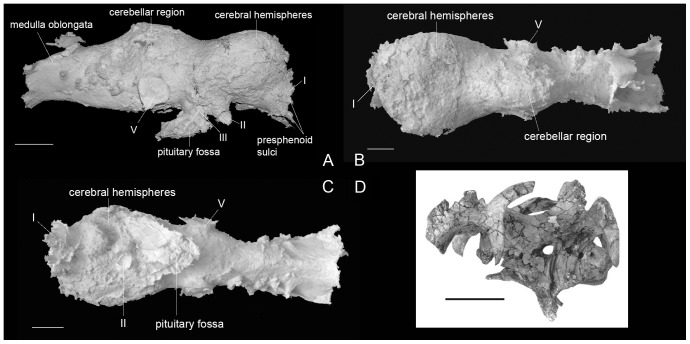
Endocranial cast of *Amurosaurus riabinini* (IRSNB R 279). (A) right lateral view, (B) dorsal view, (C) ventral view, (D) left lateral view of the braincase (AEHM 1/232) (after [2]). Roman numerals refer to cranial nerves. Scale bar equals 2 cm for (A), (B) and (C) and equals 10 cm for (D).

The general aspect of the endocast is described from the specimen IRSNB R 279 (Fig. 1). This specimen is a complete and fused braincase from a large and presumably fully grown individual [2]. The cast measures 154 mm from the base of olfactory tract to the caudal branch of the hypoglossal nerve, and has a total volume of 290 cm^3^. A larger volume for the same endocast was given in Saveliev et al. [18] (370–400 cm^3^), although those authors did not explain how this number was obtained. The maximal height of the endocast is 65 mm, excluding the pituitary body. The olfactory tract is placed rostroventral to the hemispheres. It was not possible to obtain a cast of the olfactory bulbs, although it is possible to observe that the olfactory tract is very broad, measuring 281.5 mm wide. On the edge of the tract, the bases of presphenoid sulci (Fig. 1A) can be discerned, as observed by Evans [33] on other hadrosaurid specimens.

**Figure 2:**
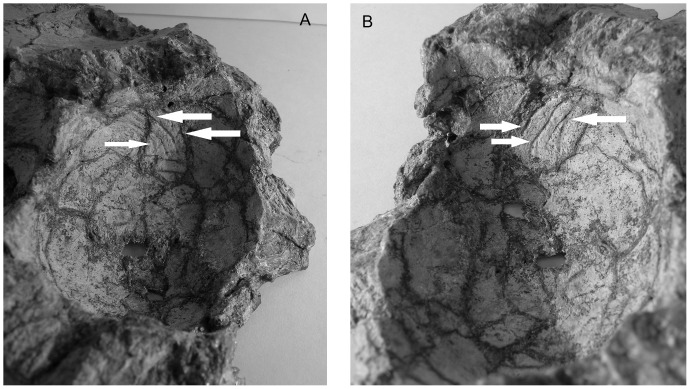
Internal wall of a frontal of *Amurosaurus riabinini* (AEHM 1/240). (A) left internal view, (B) right internal view. The specimen is 9.5 cm wide. Arrows point to some of the valleculae.

The cerebral hemispheres are rounded and wide (69 mm above the optic nerves), representing the broadest part of the brain. They are slightly compressed dorsoventrally. The large size of this region is reflected in the bones of the roof of the skull, the frontals having a domed appearance as it is usually observed in lambeosaurines [2]. As observed on AEHM 1/240, valleculae are present on the rostral part of the endocast, on the cerebral hemispheres (Fig. 2). The brains of dinosaurs are generally thought to have been separated from the endocranial walls by the intercession of cerebrospinal fluid between the meninges and/or venous sinuses within the dura [8], [13], [19], [20], [25], [34], [35]. The presence of valleculae on the endocranial surfaces of braincase bones is generally thought to indicate regions where the dural envelope was thin and that the endocranium closely reflects brain morphology in the regions where they occur [13], [14], [15], [16]. Evans [17] presented data regarding the occurrence of complex endocranial vascular impressions in hadrosaurid and pachycephalosaurid ornithischians, leading to the conclusion that the brain may have been particularly closely associated with the endocranium in the anterior and ventral regions of the brain. Valleculae have also been observed in the basal hadrosauroid *Batyrosaurus rozhdestvenskyi* [36], demonstrating that this condition was not restricted to derived lambeosaurines. The presence of the valleculae indicates that in *Amurosaurus riabinini* at least the cerebral hemispheres were in close contact with the inner wall of the braincase, and that this part of the brain is clearly represented by the endocast. The cerebral hemispheres represent 30% of the total volume of the endocast.

The endocranial cavity is nearly straight due to the extreme reduction of the cranial and pontine flexures. With its straight endocranial cavity, *Amurosaurus riabinini* shares the derived condition observed in other hadrosaurids and in *Iguanodon bernissartensis* Boulenger 1881 [37], [38]. According to Hopson [13] and Giffin [39], the most likely causes of variation in the angles of the primitive flexure pattern are absolute skull size and relative eye size. Larger genera and individuals tend to have less flexed brains than do smaller genera and individuals because of the negative allometry of the brain and eye size in reptiles [13]. In large animals such as *Amurosaurus riabinini* [40], the brain was therefore less constrained by space limitation.

The endocast considerably narrows caudal to the cerebral hemispheres. The midbrain and hindbrain are marked by a peak that is slightly lower than the cerebral hemispheres. The position of the inner ear is marked by a profound constriction behind these parts. The pituitary fossa lies posteroventral to the optic nerve. It is 24.9 mm wide and 290 mm long. As observed on the cast of AEHM 1/233, the internal carotid arteries enter the pituitary fossa posteriorly. On the endocasts of the basal ornithopods *Dryosaurus, Hypsilophodon*, and *Zephyrosaurus* [41], the pituitary body appears relatively smaller. By contrast, on hadrosaurid specimens [26], [42], [43], the pituitary body appears relatively large in comparison with the rest of the endocast. For example, Ostrom [43] assessed the pituitary body of *Kritosaurus* at 40 mm long and nearly 30 mm in height and width.

**Figure 3:**
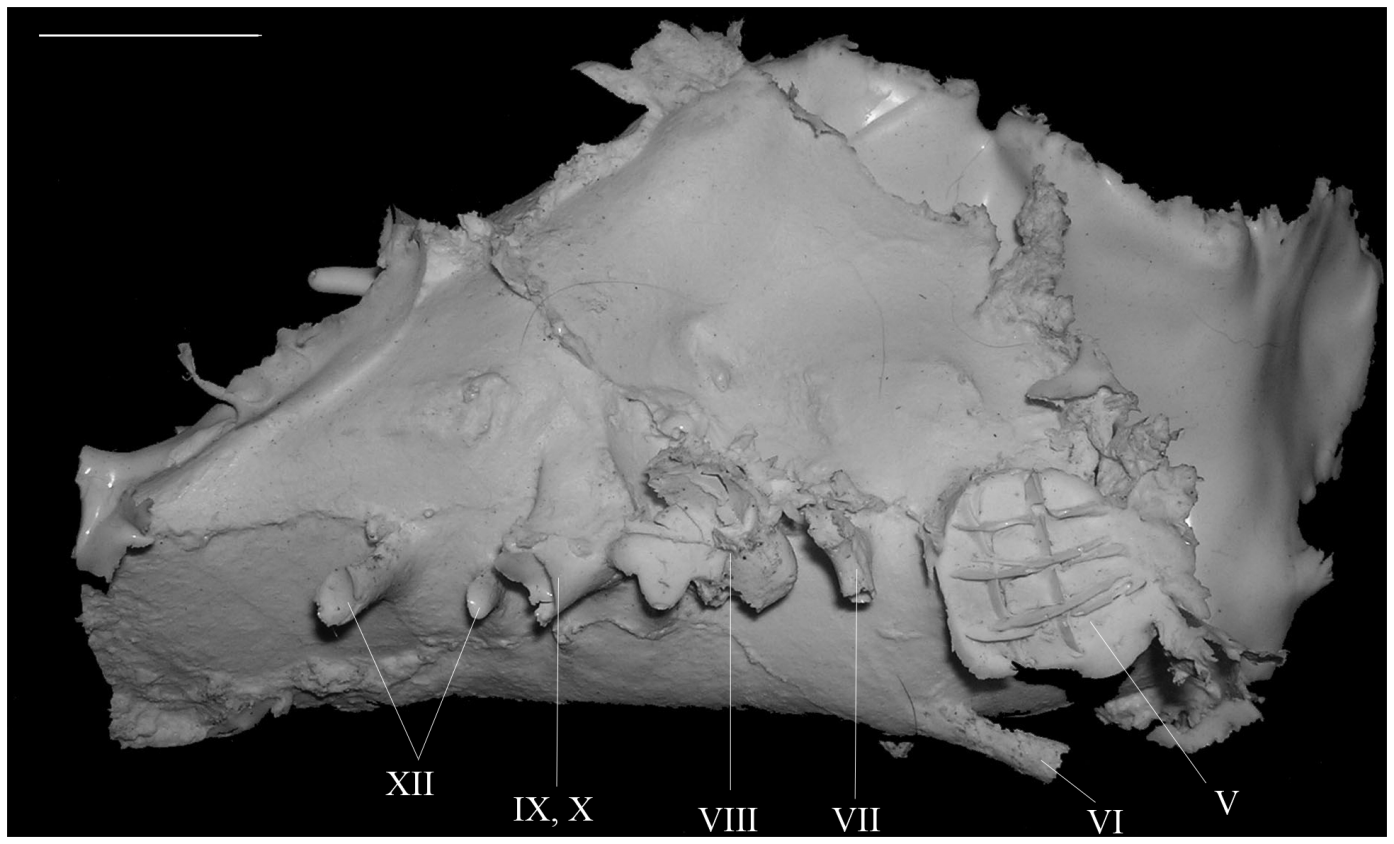
Right lateral view of the endocranial cast of *Amurosaurus riabinini* (IRSNB R 281). Roman numerals refer to cranial nerves. Scale bar equals 2

**Figure 4:**
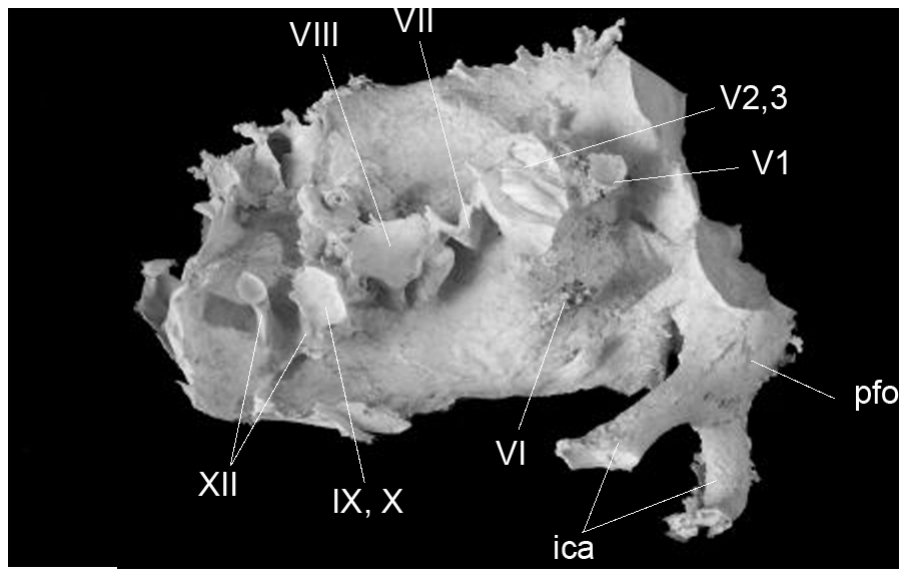
Right lateral view of the endocranial cast of *Amurosaurus riabinini* (IRNSB R 280). *Abbreviations*: *ica*, internal carotid arteries; *pof*, pituitary fossa. Roman numerals refer to cranial nerves. Scale bar equals 2 cm.

The cranial nerves (CN) are well represented on the specimens IRSNB R 281 (Fig. 3) and IRSNB R 280 (Fig. 4). The casts are roughly of the same size as IRSNB R 279 but are from incomplete braincases.

As noted above, the olfactory system is not completely preserved. The olfactory nerve (CN I) is short but large (281.5 mm wide) and lined by discrete presphenoid sulci (Fig. 1A). The number of sulci cannot be determined with precision. The position of the olfactory nerve is dorsofrontal to the cerebral hemispheres, resembling the condition observed in derived lambeosaurines, contrasting with the low position observed in hadrosaurines. It was not possible to make a cast of the olfactory bulbs. As noted by Evans et al. [26], the olfactory system of hadrosaurids was relatively smaller than in most others dinosaurs [13].

The optic nerve (CN II) exits the braincase via a large foramen in the parasphenoid caudoventrally to the cerebral hemispheres. A protrusion beneath the hemispheres represents the optic nerves that quickly diverge from each other. The width of each optic nerve is 7.5 mm. The oculomotor nerve (CN III) exits the oculomotor foramen together with the abducens nerve (CN VI) (contra Saveliev et al. [18]) caudal to the foramen for the optic nerve foramen and dorsal to the pituitary body. The foramen is formed by the parasphenoid and the laterosphenoid [2].

The trigeminal nerve (CN V) is located on the ventrolateral part of the high peak between the midbrain and the hindbrain. It extends laterally via a characteristically large, laterally expanding, funnel-shaped foramen. The large diameter of the external trigeminal foramen, 18 mm in IRSNB R 279 and in IRSNB R 280, suggests that it housed the trigeminal ganglion [43], from which the *ramus ophthalmicu*s (CN V_1_) extends rostrally via an horizontal and rostral sulcus on the laterosphenoid, and the maxillary and mandibular branches (CN V_2–3_) extend ventrally at a right angle to the *ramus ophthalmicus*.

The abducens nerve (CN VI) exits the pituitary body caudally to enter the endocranial floor at the rostral end of the medulla, behind the foramen of the trigeminal nerve. The facial nerve (CN VII) exits the endocranial cavity through the prootic between the trigeminal foramen and *fenestra vestibulari*. It diverges before reaching the lateral wall into a dorsocaudal branch (*ramus hyomandibularis*) and the ventrorostral branch (*ramus palatinus*) (contra Saveliev et al. [18]).

We also disagree with the interpretation of Saveliev et al. [18] of the vestibulocochlear nerve (CN VIII) as being small and indicating poorly developed hearing in *Amurosaurus*. Semicircular canals could not be moulded and the interpretations about the movements and habits are speculative. Considering that the group typically possesses well-developed cranial crests devoted to oral communication, it would be highly unusual if a poor sense of hearing was present in lambeosaurine dinosaurs.

The glossopharyngeal nerve (CN IX) exits the braincase through the metotic foramen immediately caudal to the *fenestra ovalis* [44]. The vagus nerve (CN X) exits immediately caudal to the glossopharyngeal nerve and is large and oval shaped. The accessory nerve (CN XI) is difficult to locate on the endocasts and its position has been discussed in the past. Ostrom [43] interpreted it as completely independent of the vagus nerve (CN X) on a *Kritosaurus* cast. Galton [41] and Evans et al. [26] considered that the accessory nerve may have exited the braincase along with the vagus (CN X) or the glossopharyngeal nerve (CN IX) through the metotic foramen. The hypoglossal nerve (CN XII) is represented by two branches. The caudal branch passes caudolaterally through the exoccipital near the occipital condyle. The rostral and smaller branch extends slightly ventrally of the medulla to a point immediately caudal to the vagus nerve (CN X). As expected in lambeosaurines [26], [44], the most caudal foramen for the hypoglossal nerve is the largest.

### Encephalization Quotient

With a circumference of 400 mm (AEHM 1/1002; length = 1120 mm), and using the hypothesis that hadrosaurids were at least occasionally bipeds, we estimated the mass of an adult *Amurosaurus* using the formula of Anderson et al. [45] at 2.03 tons. REQ was based on the assumption that the brain of *Amurosaurus* filled approximately 60% of the endocranial cavity. Consequently the estimate of the REQ is 3.8.

Because hadrosaurids are generally thought to be facultatively bipedal rather than true bipeds [46], [47], the estimation for a quadrupedal stance was also calculated, using a large humerus (AEHM 1/997; circumference = 267 mm) and the femur AEHM 1/1002 and the new formula established by Campione and Evans [23]. The mass estimation is 4.79 tons for an adult *Amurosaurus*. In this case, the REQ is 2.3.

## Discussion

The REQ (2.3–3.8) estimated for *Amurosaurus* is higher than most extant non-avian reptiles [14], as well as sauropod (*Diplodocus*, 0.53–0.69; *Nigersaurus*, 0.4–0.8; [48], [49]) and ceratopsian (*Psittacosaurus*, 1.7; *Triceratops*, 0.7; [49], [50]) dinosaurs. The REQ overlaps those of non-hadrosaurid iguanodontians (*Iguanodon bernissartensis*, 1.88–3.14; *Mantellisaurus atherfieldensis*, 1.68–2.67; [38]) and is similar to those calculated for hadrosaurine hadrosaurids (2.8; [26]) and for the lambeosaurine hadrosaurid *Hypacrosaurus altispinus* (2.3–3.7; [26]). Estimated REQ value for *Amurosaurus* also appear lower than most non-avian theropods (*Ceratosaurus*, 3.31–5.07; *Allosaurus*, 2.4–5.24; *Acrocanthosaurus*, 2.75–5.92; *Citipati* 3.6; *Tyrannosaurus*, 5.44–7.63; *Troodon*, 7.76; [48]).

Edinger [51], [52] detailed evidence that the gigantism observed in many fossil species might be correlated to hyperpituitarism. Hyperpituitarism is a well-known condition with several manifestations such as acromegaly and diverse pathologies [53], [54], [55], [56], [57]. It is possible that the great sizes and heavy body masses of some dinosaurs were tied to an enlargement of the pituitary gland, which led to increased production of growth hormone. It seems that dinosaurs achieving large size, such as *Amurosaurus riabinini*, were also characterized by a large pituitary fossa. The pituitary gland of large sauropods [58], [59], [60] is indeed relatively large compared to the size of the brain. This hypothesis requires, however, further testing and quantification.

We disagree with the interpretation of Saveliev et al. [18] that *Amurosaurus* had small, slow-moving eyeballs. The optic lobes are not apparent on the endocranial cast, and are also not apparent on the casts of other lambeosaurines [26], [33], [44] and in extant crocodiles (personal observation). Crocodiles have excellent eyesight [61], [62]. As previously noted, the brain of *Amurosaurus* was not constrained by space limitation. Connective tissues probably covered the optic lobes, preventing their appearance on the cast. As a results, it is not possible to assess the size of the optic lobes, but there is no reason to consider that *Amurosaurus* had peculiarly small eyes. The size of the orbits is in the same range as that observed in other hadrosaurids. The diversity of cranial crests exhibited by lambeosaurines and the presence of sexual dimorphism [63], [64] would favor the hypothesis of animals using visual cues as means of communication. This hypothesis has been often discussed and is currently widely accepted [13], [33], [47], [64]. Saveliev et al. [18] hypothesized that the vomeronasal system played a role in the reproduction of hadrosaurids, even though this organ is absent in all extant archosaurs. The presence of the vomeronasal system in *Amurosaurus* is thus ruled out [65]. *Amurosaurus* was a strict and specialized herbivore [47], [66] that lived in a savannah-like environment with oasis vegetation along the banks of lakes and rivers, under a warm-temperate and relatively arid climate [2].

The cerebral hemispheres of *Amurosaurus riabinini* were slightly flattened and relatively smaller than those in more derived North American lambeosaurines [26]. The shape and the relative size of the cerebral hemispheres reflect the phylogenetic position of *Amurosaurus riabinini* as a basal member of the Lambeosaurinae ([2], [7], [44], contra [67]).

According to Evans et al. [26], the most striking aspect of the brain endocast of lambeosaurine hadrosaurids is the relatively large size of the cerebrum. The estimated relative volume of the cerebrum (CRV = cerebrum volume/endocast volume) in four late Campanian lambeosaurines from North America varies between 35 and 42% [26]. The cerebrum of lambeosaurines is therefore larger than that of large theropods such as *Carcharodontosaurus* (24%) and *Tyrannosaurus rex* (33%), but compares favorably with the maniraptoran theropod *Conchoraptor* (43%) and even with the basal bird *Archaeopteryx* (45%). With a CRV of 30%, *Amurosaurus* is slightly under the estimated values for North American lambeosaurines but above those for the non-hadrosaurid iguanodontians *Iguanodon bernissartensis* (19%) [38] and *Lurdusaurus arenatus* Taquet and Russell, 1999 [68] (19%, [38]).

**Figure 5:**
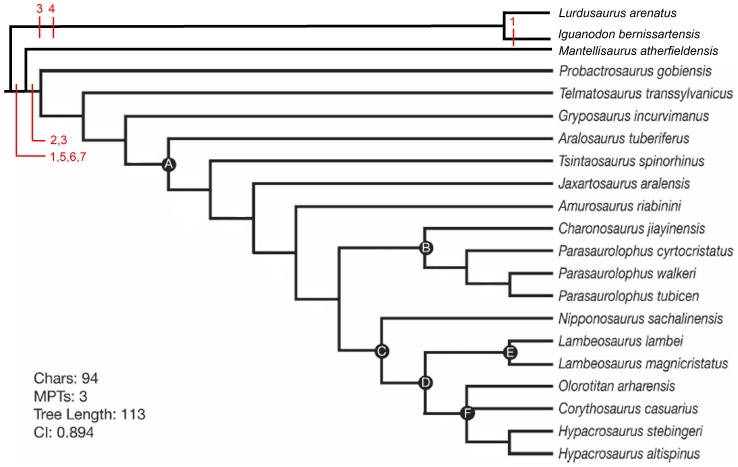
Strict consensus of three most parsimonious trees recovered in phylogenetic analysis of Lambeosaurinae. Strict consensus tree showing the phylogenetic relationships of *Amurosaurus riabinini* with other specimens discussed. Numbers correspond to endocranial characters: 1, width of olfactory peduncle; 2, volume of the pituitary gland; 3, absence of cranial and pontine flexures; 4, presence of the floccular fossa; 5, CRV; 6, REQ; 7, maximal width cerebral hemispheres/total length brain (modified from [76]).

The presence of an enlarged brain and cerebrum relative to body size is usually equated with increased behavioral complexity in vertebrates [14], [19], [20], [25]. Individuals living in groups are subjected to social interactions that require rapid and elaborate feedback to maintain the social hierarchy and the reproductive fitness of the individual among his group [69], [70], [71], [72]. Dunbar [73] hypothesized that brain size can be a reliable estimator of group size because of the potential close relationship between neocortex size, brain cognitive capacity and individual recognition. The relatively large size of the brain and the cerebrum in lambeosaurines is consistent with the range and complexity of social behaviors inferred from the hypothesis that the supracranial crest was an intraspecific signaling structure for visual and vocal communication [26], [74]. However, a similar increase in the relative size of the cerebellum can be observed in *Mantellisaurus atherfieldensis* (Hooley, 1925) [75] and in more basal Iguanodontia. The mix of ancestral and more derived characters exhibited by *Amurosaurus riabinini* is interpreted here as a reflection of its intermediate position in the phylogeny of the Lambeosaurinae (Fig. 5).
